# The functional ovarian anatomy of 492 women aged 18–22 years: a population-based study in Norway

**DOI:** 10.1093/hropen/hoaf057

**Published:** 2025-09-24

**Authors:** Mari Landås Warp, Karoline Hansen Skåra, Thea Karoline Grindstad, Kirstine Kirkegaard, Nils-Halvdan Morken, Cecilia Høst Ramlau-Hansen, Liv Bente Romundstad, Siri Eldevik Håberg, Hans Ivar Hanevik

**Affiliations:** Fertilitetsavdelingen Soer, Telemark Hospital Trust, Porsgrunn, Norway; Centre for Fertility and Health, Norwegian Institute of Public Health, Oslo, Norway; Department of Clinical Science, University of Bergen, Bergen, Norway; Centre for Fertility and Health, Norwegian Institute of Public Health, Oslo, Norway; Centre for Fertility and Health, Norwegian Institute of Public Health, Oslo, Norway; Department of Gynecology and Obstetrics, Aarhus University Hospital, Aarhus, Denmark; Centre for Fertility and Health, Norwegian Institute of Public Health, Oslo, Norway; Department of Clinical Science, University of Bergen, Bergen, Norway; Department of Obstetrics and Gynecology, Haukeland University Hospital, Bergen, Norway; Department of Public Health, Aarhus University, Aarhus, Denmark; Centre for Fertility and Health, Norwegian Institute of Public Health, Oslo, Norway; Volvat Spiren Fertility Clinic, Trondheim, Norway; Centre for Fertility and Health, Norwegian Institute of Public Health, Oslo, Norway; Department of Global Public Health and Primary Care, University of Bergen, Bergen, Norway; Fertilitetsavdelingen Soer, Telemark Hospital Trust, Porsgrunn, Norway; Centre for Fertility and Health, Norwegian Institute of Public Health, Oslo, Norway

**Keywords:** ovarian function, young adulthood, The Norwegian Mother, Father and Child Cohort Study, MoBa daughters, AMH/anti-Müllerian hormone, AFC/antral follicular count

## Abstract

**STUDY QUESTION:**

How do measures of functional ovarian anatomy (ovarian volume, antral follicle count, endocrinological profile) vary among women between 18 and 22 years?

**SUMMARY ANSWER:**

We found considerable inter-individual variability in functional ovarian anatomy in young adult females examined after puberty but before the age-related decline in ovarian function sets in.

**WHAT IS KNOWN ALREADY:**

Functional ovarian anatomy varies with age and disease. Fecundability in healthy females peaks in early adulthood when puberty is completed and the age-related decline in ovarian function is insignificant.

**STUDY DESIGN, SIZE, DURATION:**

Daughters born into a population-based pregnancy study, The Norwegian Mother, Father and Child Cohort Study (MoBa) were examined on menstrual cycle days 2–5. Recruitment to this cross-sectional study started in August 2021 and is ongoing.

**PARTICIPANTS/MATERIALS, SETTING, METHODS:**

The 492 participants were aged 18–22 years and were not using hormonal contraceptives when they underwent a clinical examination during the early follicular phase of their menstrual cycle. Another group of 8146 MoBa daughters who were of similar age and who answered a questionnaire were studied to assess representativeness. Participants contributed with fasting blood samples, anthropometric measurements, and a questionnaire. Trained clinicians performed a transvaginal ultrasound to assess ovarian anatomy.

**MAIN RESULTS AND THE ROLE OF CHANCE:**

The interquartile range was 9.3–17.2 cm^3^ for total ovarian volume, 21–37 for total antral follicle count (AFC), and 16.0–35.4 pmol/l for serum anti-Müllerian hormone (AMH). We found positive correlations between ovarian volume and AFC (r = 0.52, *P* < 0.01), between ovarian volume and AMH (r = 0.53, *P* < 0.01), and between AFC and AMH (r = 0.71, *P* < 0.01). Participants’ mean left ovarian volume (6.5 cm³, 95% CI: 6.1–6.8) and mean right ovarian volume (7.4 cm³, 95% CI: 7.1–7.8) differed (t(446) = 4.8, *P* < 0.001). The examined population was representative of the broader MoBa daughters population.

**LIMITATIONS, REASONS FOR CAUTION:**

The study is ongoing and has a low participation rate possibly due to the intimate nature of the clinical examinations.

**WIDER IMPLICATIONS OF THE FINDINGS:**

There are large differences between young women in terms of functional ovarian anatomy. Follow-up of reproductive outcomes for these women, with linkage to the medical birth registry of Norway, could detect early signs of reduced fertility already in young adulthood.

**STUDY FUNDING/COMPETING INTEREST(S):**

This study was funded by the Norwegian Institute of Public Health, Oslo, Norway, and by Telemark Hospital Trust, Porsgrunn, Norway, and was partly supported by the Research Council of Norway through its centres of excellence funding scheme, project number 262700 and project no. 320656. The project was co-funded by the European Union (EU) (European Research Council (ERC), BIOSFER, 101071773). Views and opinions expressed are however those of the author(s) only and do not necessarily reflect those of the EU or the ERC. Neither the EU nor the granting authority can be held responsible for them. The authors report no competing interests.

**TRIAL REGISTRATION NUMBER:**

N/A.

WHAT DOES THIS MEAN FOR PATIENTS? Female fertility is declining, making early preventive measures increasingly important. To diagnose abnormalities, it is essential to first establish what is considered normal. Few previous studies have investigated the ovaries of young women after puberty and before having children, without the influence of hormone birth control and without selection based on previous disease or infertility. This study is the first to report data on ovarian volumes, number of follicles (small sacs that each holds an oocyte), and hormonal levels in daughters born into a large Norwegian birth cohort. Although the examined daughters were also representative of the broader cohort population, the findings reveal a large variation in how the ovaries look and function, including a left–right size difference. Follow-up of the reproductive outcomes for these women could detect early signs of reduced fertility already in young adulthood.

## Introduction

Ovarian anatomy and function vary with female age. Ovarian volume is small (<2 cm^3^) in prepubertal girls (Gilligan *et al.*, 2019) and elderly women (Pavlik *et al.*, 2000), reflecting low ovarian function in those phases of life. Ovaries are normally larger (2–10 cm^2^) and more physiologically active between puberty and menopause and reach their peak volume at around 20 years of age (Kelsey *et al.*, 2013). The ovarian volume comprises stromal tissue and follicles, and the functional ovarian reserve is described as the pool of growing follicles in the ovary (follicles of 2–5 mm in diameter), from which one follicle is usually selected for ovulation (Moolhuijsen and Visser, 2020). Another aspect of functional ovarian anatomy is the antral follicle count (AFC), comprising follicles of 2–10 mm in size (Bentzen *et al.*, 2013). The granulosa cells of antral follicles produce and secrete anti-Müllerian hormone (AMH) (Weenen *et al.*, 2004; Jeppesen *et al.*, 2013). Previous studies showed associations between ovarian volume and both AFC levels and serum levels of AMH (Zhu *et al.*, 2016; Korsholm *et al.*, 2017), though associations are not consistent (Alebic *et al.*, 2018).

Early adulthood (18–25 years) is when ovarian volume, AFC, and AMH reach their peak levels in population-based studies (Kelsey *et al.*, 2011, 2013; Jain *et al.*, 2023). Most of the biological processes associated with puberty have been completed by this stage, while the age-related decline in AFC and AMH still exerts little influence on ovarian function. At this age, the proportion of euploid oocytes released from the ovary is high (Pellestor *et al.*, 2006), and early adulthood is when females are most fecund (Dunson *et al.*, 2002; Susiarjo *et al.*, 2007). Early adulthood is an age when important decisions are taken concerning both initial phases of family formation and other lifestyle aspects that may impact a woman’s fertility. Studying the functional ovarian anatomy (ovarian volume, AFC, endocrinological profile) in a large population-based cohort of women in early adulthood will increase the understanding of normal variation in this important phase of the ovarian lifespan. Further, examining ovaries at their presumed peak size and functional capacity could be valuable in the search for potential predictors of relevant diseases with later onset, such as premature ovarian insufficiency and infertility. Notwithstanding, previous studies of ovarian volume have focused on observations in women above 25 years of age (Pavlik *et al.*, 2000; Jokubkiene *et al.*, 2012; Korsholm *et al.*, 2017) or observations in girls from birth to puberty (Lie Fong *et al.*, 2012; Gilligan *et al.*, 2019; Fischer *et al.*, 2024). Only a few studies with low number of participants have investigated ovarian volume (Kelsey *et al.*, 2013) and other aspects of functional ovarian anatomy (Kelsey *et al.*, 2011) in women in early adulthood. In particular, data are lacking from women in early adulthood who are not currently using hormonal contraceptives (Landersoe *et al.*, 2020).

Our main aim in this study was to assess the functional ovarian anatomy during early adulthood in women who did not use hormonal contraceptives at the time of examination. We describe variations in ovarian volume, AFC, and AMH in these women, and assess how these measures correlate. Participants in the study were 492 women, aged 18–22 years, who were born into the Norwegian Mother, Father and Child Cohort study (MoBa) between 1999 and 2005 and who currently participate in an ongoing sub-study of MoBa that comprises clinical and gynaecological examinations. The secondary aim was to investigate the representativeness of the 492 participants with regards to the broader MoBa daughter population.

## Materials and methods

MoBa is a large pregnancy cohort conducted by the Norwegian Institute of Public Health (NIPH). Pregnant women from across Norway were invited to participate in their routine ultrasound appointment during the recruitment period between 1999 and 2008 (Magnus *et al.*, 2016). About 95 200 mothers and 75 200 fathers were recruited, and 114 500 children were born into the cohort. Women and their partners could participate with subsequent pregnancies during the inclusion period, and MoBa mothers and fathers answered multiple questionnaires from pregnancy, after their child was born, and until today. The MoBa children have also been followed up with questionnaires answered by their parents at first, then later by themselves from around 13 years of age, and until the present. In the current study, information on socioeconomic status in childhood comes from a questionnaire answered by the MoBa mothers and fathers during pregnancy with the child in question and was based on the parents’ highest completed or ongoing education level (<high school, high school, up to 4 years of college, >4 years of college). Information about MoBa daughters’ gestational length at birth, birthweight, and their mother’s parity was collected through linkage to the Medical Birth Registry of Norway (MBRN). The establishment of MoBa and initial data collection was based on a license from the Norwegian Data Protection Agency and approval from The Regional Committees for Medical and Health Research Ethics. Currently, the MoBa cohort, including the data collection for this study, is regulated by the Norwegian Health Registry Act. This study was approved by The Regional Committees for Medical and Health Research Ethics of South/East Norway, project number 277291.

### MoBa daughters undergoing clinical examinations

MoBa daughters aged >18 years and 3 months were invited to a clinical examination at one of two study clinics (Telemark Hospital Trust, Porsgrunn, Norway or Volvat Spiren, Trondheim, Norway). Recruitment started in August 2021 and is ongoing. Clinical data in this study are from November 2021, when the first participant was examined, to September 2024. Within this period, a total of 21 542 MoBa daughters were invited to participate, and so far, 492 have undergone examination. After written consent, participants were contacted via mobile phone by personnel from the respective study clinic. According to study protocol established prior to invitation, inclusion criterion was availability for examination between Days 2 and 5 during the menstrual cycle. Participants currently using hormonal contraceptives had to discontinue the use, and the earliest possible time for examination was between Days 2 and 5 during their second menstrual cycle after discontinuing the hormonal contraceptives. Participants with oligomenorrhea or amenorrhea (not due to pregnancy) were examined on any day after ≥60 days of no menstruation.

### Clinical examination

Participants arrived at the clinic fasting from midnight the day before (minimum 8 h). First, height, hip, and waist circumferences were measured in cm. A bioelectrical impedance analyser (TANITA DC-240MA; Tanita Corporation, Tokyo, Japan) was used to measure body weight, lean muscle mass and bone mineral mass (kg), body fat (%), total body water (%), and visceral fat level (indicated as a number between 1 and 59). Blood samples were taken while still fasting. Participants answered a self-administered questionnaire, and clinical personnel inquired about menstrual cycle regularity, bleeding duration, and menstrual pain. A urine sample was collected at any time during the visit. Measurements of the right hand’s digit lengths in mm were also conducted. We recorded an electrocardiogram and measured blood pressure three consecutive times using an automatic blood pressure monitor. Genital examination was preformed, including measurements of anogenital distances. By transvaginal ultrasound, we measured uterine volume (length, anterior–posterior diameter, and width), cervical length, endometrial thickness, ovarian volume (length, width, height), and the size and number of all follicles on both ovaries (from 2 to 25 mm in intervals of 2 mm, i.e. 2–4 mm, 5–7 mm, and so forth). Antral follicles were defined as follicles of 2–10 mm. Pathologies of the internal genitalia were documented if found (i.e. ovarian cysts or follicles >25 mm, absence of sliding sign, intrauterine polyps, myomas). Participants with pathological findings were informed and referred for follow-up. All ultrasound scans were conducted by one of four trained clinicians. The measurements were standardized by study protocol. The ultrasound machines used were GE Voluson E8 with transducer RIC5-9-D (4-9MHz) or GE Voluson S8 with transducers E8C-RS (4–10 MHz) or RIC5-9A-RS (4–10 MHz), (GE Healthcare, Chicago, IL, USA).

### Biochemical analyses

Blood samples were drawn and sent the same day to a central laboratory (St Olav’s Hospital Trust Trondheim, Norway) for analysis within 24 h. We analysed serum levels of AMH, FSH, LH, oestradiol, testosterone, sex hormone-binding globulin (SHBG), prolactin, thyroid-stimulating hormone, free thyroxine, insulin c-peptide, insulin-like growth factor 1, iron, total iron-binding capacity, ferritin, creatinine, albumin, C-reactive protein, total cholesterol, low-density lipoprotein cholesterol, high-density lipoprotein cholesterol, and triglycerides. Haemoglobin and haemoglobin A1c were measured from full blood.

Serum level of AMH (pmol/l) was measured on the Cobas pro e801 (Roche Diagnostics, Basel, Switzerland), using an electrochemiluminescence immunoassay (CLIA), with an intra- and inter-assay coefficient of variation (CV) of 1.9%, and lower limit of detection (LOD) of 0.1 pmol/l. FSH and LH (IU/l) were measured on the Siemens Atellica IM1600 (Siemens Healthineers, Erlangen, Germany) using CLIA, with an intra- and inter-assay CV of 5.4%, and 3.4%, and a LOD of 0.8 and 0.2 IU/l, respectively. Testosterone (nmol/l) was measured on the Agilent 6465 Triple Quad LC/MS–MS (Agilent Technologies, Santa Clara, CA, USA) using High Pressure Liquid Chromatography (HPLC), with an intra- and inter-assay CV of 6.0% and LOD of 0.1 nmol/l. Oestradiol (nmol/l) was measured on the Siemens Atellica IM1600 using CLIA with a CV of 11.0% and LOD of 0.1 nmol/l. If oestradiol could not be detected, the analysis was repeated on the Agilent 6465 Triple Quad LC/MS–MS using HPLC, with a LOD of 0.01 nmol/l. SHBG (nmol/l) was measured on the Siemens Atellica IM1600 using CLIA with an intra- and inter-assay CV of 4.5% and LOD of 2.0 nmol/l. Samples of full blood, plasma, and urine were stored at the clinics at −20°C for up to 7 days before being shipped on dry ice to the NIPH’s Biobank, where they are currently stored at −80°C.

### Statistical analyses and cohort representability

We calculated ovarian volumes (cm^3^) on the left and right ovaries using the prolate ellipsoid formula (height × width × length ×0.523) (Pavlik *et al.*, 2000). Total ovarian volume was calculated by adding the left and right ovarian volumes when measurements from both ovaries were available, excluding participants who had measurements from only one side. Mean ovarian volume was calculated by dividing total ovarian volume by two for those with measurements from both sides or simply using the left or right ovarian volume for those with measurements from only one side. Total AFC was calculated by adding the count from left and right ovaries for women with measurements from both sides, excluding participants who had measurements from only one side. Mean AFC was calculated by dividing total AFC by two for those with measurements from both ovaries or simply by using the left or right AFC for those with measurements from only one side. A paired *t*-test was used to assess differences in mean ovarian volumes and AFC counts separately between the left and right sides. Using Pearson’s correlation test, we calculated the pairwise correlation between total ovarian volumes, total AFC and serum levels of AMH.

**Figure 1.**
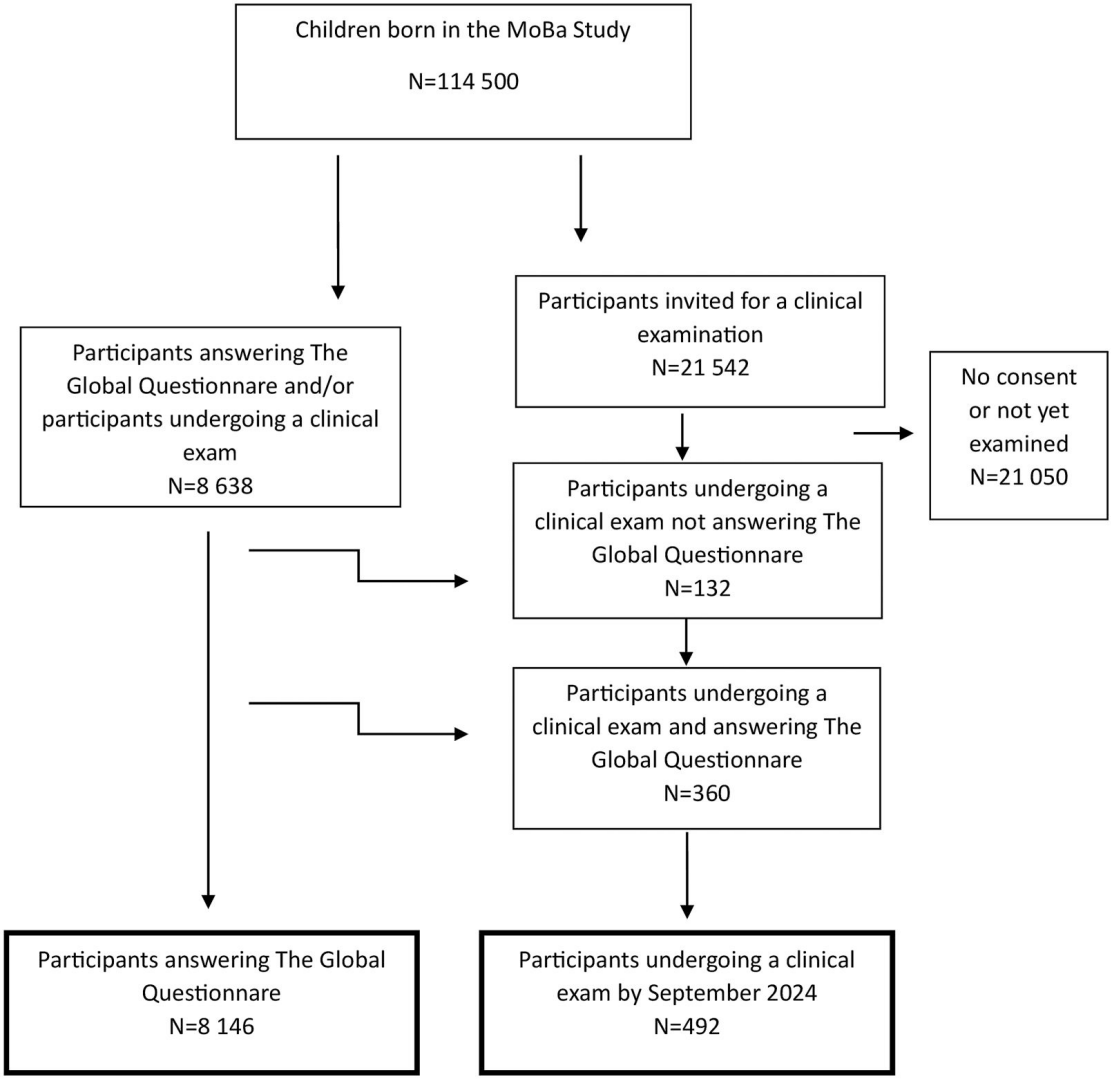
**Flow chart of study samples: daughters form the Norwegian Mother, Father and Child Cohort Study undergoing clinical examination (N = 492) and daughters from the Norwegian Mother, Father and Child Cohort Study answering the Global Questionnaire** (N = 8146).

For the secondary aim of comparing participants who underwent clinical examination (N = 492) to the MoBa cohort at large for representability, we used data from MoBa’s Global Questionnaire. Here, MoBa children >18 years of age answered questions about life choices, family, and the future in April 2024. A total of 8506 daughters answered, also yielding data about weight, height, and age at menarche. Of these, 360 had also participated in the clinical examinations. We compared background characteristics from the 8146 participants who answered the Global Questionnaire only to the 492 participants undergoing clinical examination (Fig. 1). Here, we checked variance when comparing continuous data and used a two-sided *t*-test or Welch’s *t*-test where appropriate. When comparing categorical variables, we used chi-square test of independence.

Some participants had polycystic ovarian morphology (PCOM). PCOM is defined as at least one ovary with ≥20 antral follicles and/or at least one ovary ≥10 cm^3^. In Supplementary Table S1, we compared clinical and biochemical variables between participants with and without PCOM.

All analyses were conducted in STATA version 17 (Stata Corp, College Station, TX, USA).

## Results

From the 492 participants in this study, data from the clinical examination were available for 485 participants. Data from the questionnaire answered at the study clinic were available from 491 participants, and blood test measurements were available from 439 participants. Complete datasets from clinical examination, questionnaire, and blood tests were available from 437 women. Information about day in menstrual cycle when attending the clinic was available from 483 participants, and most (419/483 = 86.7%) were examined on menstrual cycle days 2–5 as required by the study protocol. For practical reasons, 16 participants were examined on Day 1 or between Days 6–8 during their cycle. A total of 33 participants were examined on Day 60 or later (due to missing menstruation ≥60 days after invitation to the study). Some women could not be examined on any of these cycle days due to other reasons not classified (N = 15). All participants were not using hormonal contraceptives for a minimum of two menstrual cycles prior to examination, including 309 (309/491 = 63.0%) participants who had a history of using hormonal contraceptives (intrauterine device, contraceptive pill, injection, or implant). Most participants had a menstrual cycle between 24 and 35 days (368/485 = 75.9%) and between 5 and 7 bleeding days (453/485 = 93.4%) in the absence of hormonal contraceptive use. A total of 116 (116/487 = 23.8%) participants reported never having had sexual intercourse, and 13 (13/491 = 2.6%) reported having previously been pregnant, and of those, two participants had a live birth.

Table 1 presents descriptive statistics (mean, SD, median, IQR) of the participants’ functional ovarian anatomy and biochemical analyses. We excluded women with ovarian cysts or follicles >25 mm when presenting the results for ovarian volumes, AFCs, and AMH levels. We also excluded one outlier with total ovarian volume >80 cm^3^. The ovarian volume of both ovaries combined (total ovarian volume) was 13.9 cm^3^ (SD 6.3). Participants’ mean left ovarian volume was 6.5 cm³ (95% CI: 6.1–6.8) and mean right ovarian volume was 7.4 cm³ (95% CI: 7.1–7.8), a statistically significant difference (t(446) = 4.8, *P* < 0.001). The mean AFC of both ovaries combined (total AFC) was 30.8 (SD 14.4). When comparing the AFC between the left (mean 14.8 (95% CI: 14.1–15.5)) and the right (mean 16.1 (95% CI: 15.2–16.7)) ovaries, the difference in AFC between ovaries was statistically significant (t(446) = −4.1, *P* < 0.001). The participants’ mean serum AMH level was 27.4 pmol/l (SD 17.0) and ranged from 0.2 to 85.3 pmol/l. A total of 51 participants had serum AMH values ≤10.0 pmol/l.

**Table 1.** Descriptive statistics of MoBa daughters’ functional ovarian anatomy.

Clinical measures	Measure unit	n	Mean	SD	Median	IQR	***P*-value** ^4^
**Antral follicular count—left ovary** ^1^	count	456	14.8	7.4	13.0	10.0–18.0	<0.01
**Antral follicular count—right ovary** ^1^	count	461	16.1	8.1	14.0	11.0–19.0	
**Mean antral follicular count** ^1^	count	470	15.4	7.3	14.0	10.5–18.5	n/a
**Total antral follicular count** ^1^	count	447	30.8	14.4	28.0	21.0–37.0	n/a
**Ovarian volume—left ovary** ^1^ ^,^ ^2^	cm^3^	456	6.5	3.7	5.7	4.0–8.2	<0.01
**Ovarian volume—right ovary** ^1^ ^,^ ^2^	cm^3^	462	7.4	3.9	6.7	4.8–9.2	
**Mean ovarian volume** ^1^ ^,^ ^2^	cm^3^	452	7.0	3.2	6.5	4.7–8.6	n/a
**Total ovarian volume** ^1^ ^,^ ^2^	cm^3^	*447*	13.9	6.3	13.0	9.3–17.2	n/a
**Biochemical measures**	**Measure unit**	**n**	**Mean**	**SD**	**Median**	**IQR**	
**Anti-Müllerian hormone** ^1^	pmol/l	423	27.4	17.0	23.4	16.0–35.4	
**FSH** ^3^	IU/l	438	6.7	5.5	6.3	5.0–7.6	
**LH** ^3^	IU/l	438	5.8	4.5	4.6	3.6–6.6	
**Oestradiol** ^3^	nmol/l	437	0.1	0.1	0.1	0.1–0.2	
**Testosterone** ^3^	nmol/l	438	1.1	0.4	1.0	0.8–1.3	
**Sex hormone-binding globulin** ^3^	nmol/l	438	55.5	25.0	51.0	38.0–68.0	
**Free androgen index** ^3^ ^,^ ^5^		438	0.2	0.2	0.2	0.1–0.3	

1 Ovaries with cysts or follicles larger than 25 mm were excluded: 6 participants had cysts identified on the left ovary and 13 on the right ovary. In addition, one outlier is excluded due to total ovarian volume >80 cm^3^.

2 Ovarian volume in cm^3^ calculated according to the prolate ellipsoid formula: (length × width × height × 0.523).

3 Means, SD, and medians of FSH, LH, sex hormone-binding globulin, testosterone, oestradiol, and free androgen index is not excluded participants with cysts.

4 Paired *t*-test: comparison of left and right ovarian volume and left and right antral follicle count (AFC). AFC is defined as follicles between 2 and 10 mm.

5 Free androgen index=testosterone/sex hormone-binding globulin.

**Figure 2.**
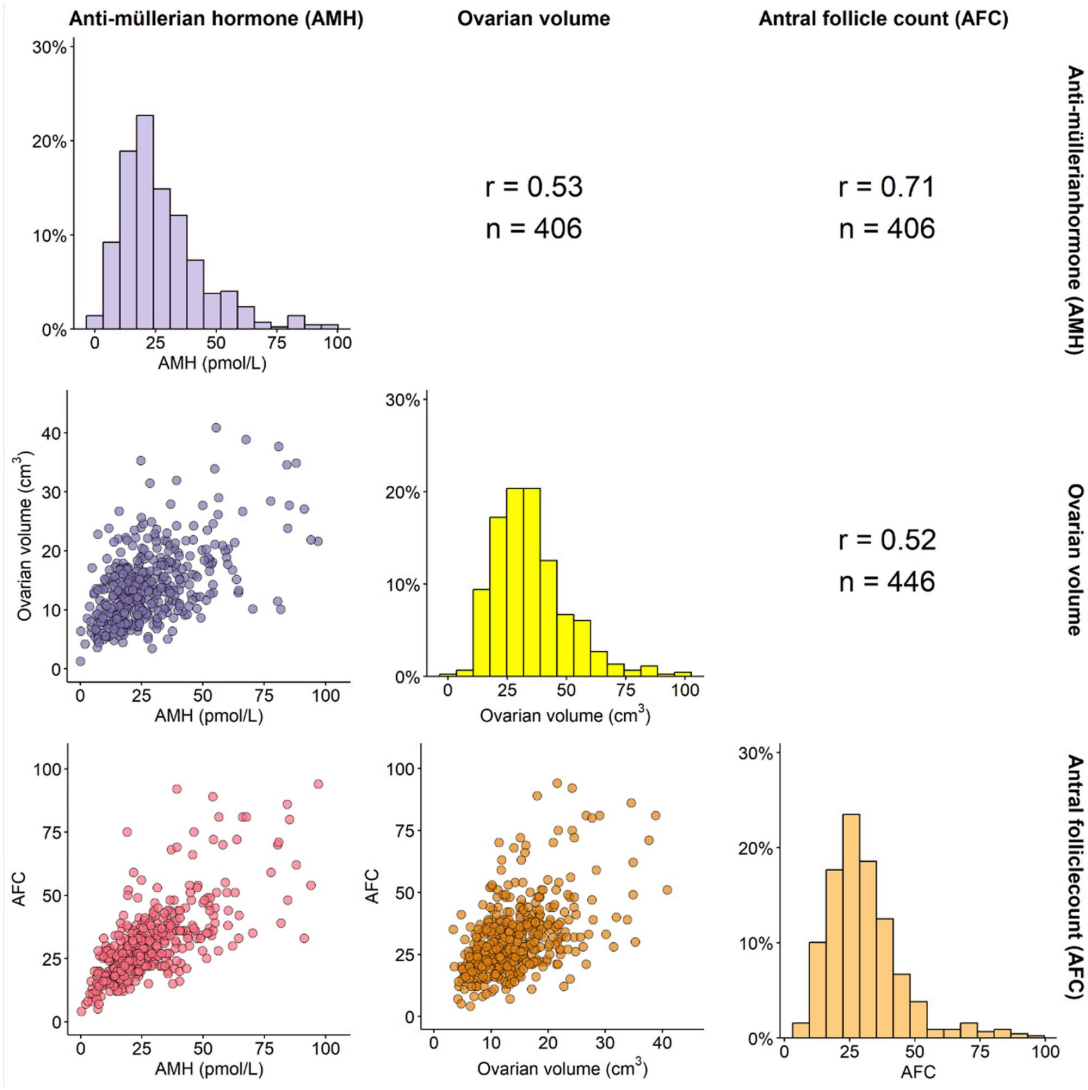
**Correlation between total ovarian volumes, total antral follicular count, and serum anti-Müllerian hormone levels.** The diagonal panels show the distributions of serum anti-Müllerian hormone levels (AMH; pmol/l; shown in light purple), total ovarian volumes (cm^3^; shown in yellow), and total antral follicular count (AFC; shown in light orange). The lower panels show the scatter plots of the pairwise relationships between AMH and total ovarian volume (shown in dark purple), AMH and AFC (shown in pink), and AFC and total ovarian volume (shown in dark orange). Pearson’s correlation test was used to generate the correlation coefficients (shown in upper panel) between total ovarian volume and total antral follicular counts (r=0.52), between total ovarian volume and AMH (pmol/l) (r=0.53), and between antral follicular count and AMH (pmol/l) (r=0.71). All correlation coefficients were statistically significant with *P*-value ≤ 0.05 (not shown).

**Figure 3.**
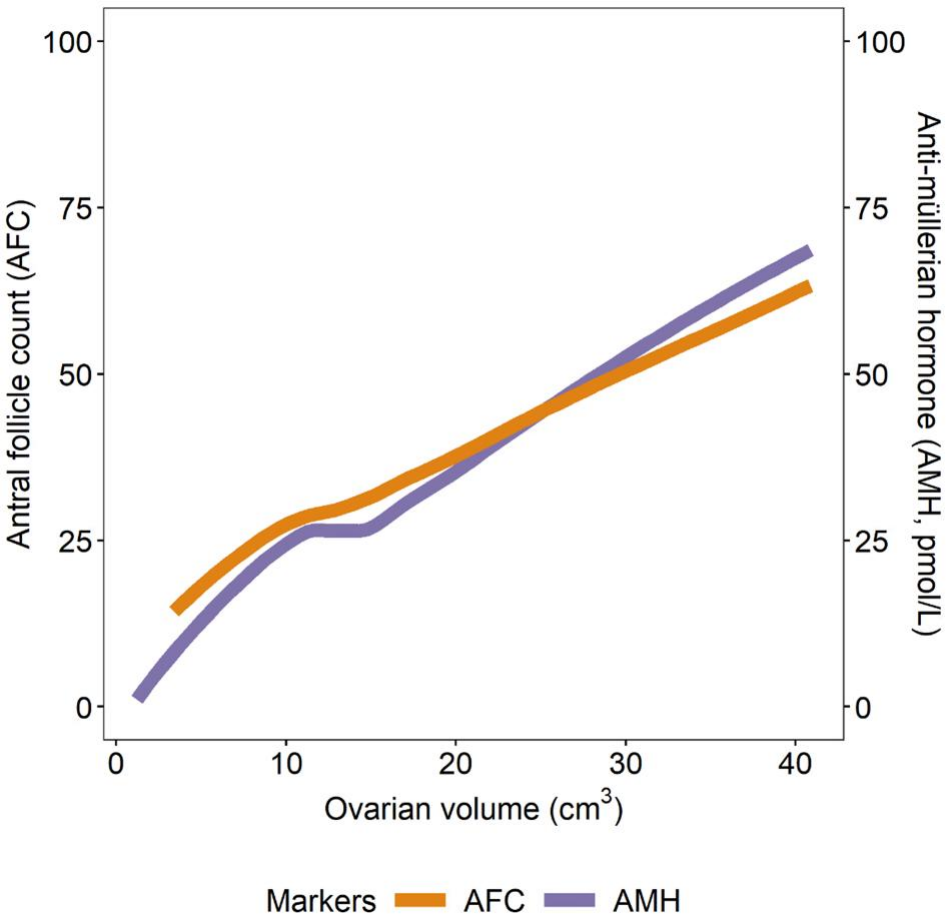
**Association between total ovarian volume, total antral follicle count, and anti-Müllerian hormone (pmol/l).** The association between total antral follicular count and total ovarian volumes (cm^3^) (N = 446) is illustrated by the orange line (primary *y*-axis), whereas the association between serum anti-Müllerian hormone levels (pmol/l) and total ovarian volumes (cm^3^) (N = 406) is illustrated by the purple line (secondary *y*-axis). *X*-axis represents the total ovarian volume in cm^3^.

Figure 2 shows the correlation coefficients between total ovarian volume and total AFC (N = 446), total ovarian volume and serum AMH (N = 406) and between total AFC and serum AMH (N = 406). The results were as follows: r = 0.52 (*P* ≤ 0.01) between total ovarian volume and total AFC, r = 0.53 (*P* ≤ 0.01) between total ovarian volume and serum AMH, and r = 0.71 (*P* ≤ 0.01) between total AFC and serum AMH. These values indicate a moderate positive correlation between the variables. Figure 3 illustrates the total ovarian volume plotted against total AFC and serum AMH levels. The figure shows that ovarian volume increases in tandem with rising AFC and serum AMH levels.

Only 25 participants in the current study with available data on both left and right ovarian volumes had a total ovarian volume of <6 cm^3^. Those with either a left ovary 1 SD smaller than the mean (2.8 cm^3^), or a right ovary 1 SD smaller than the mean (3.6 cm^3^), comprised 56 and 53 of participants, respectively, and they had a mean serum AMH of 18.8 and 17.1 pmol/l, and a mean AFC of 9.7 and 11.0, respectively.

There were 67 participants in the current study, with available data on both left and right ovarian volumes, who had a total ovarian volume of >20 cm^3^, and 127 participants had either a left or a right ovary >10 cm^3^. Those with either a left ovary 1 SD larger than the mean (10.1 cm^3^), or a right ovary 1 SD larger than the mean (11.3 cm^3^), comprised 62 and 67 participants, respectively, and they had a mean serum AMH of 38.1 and 36.2 pmol/l, and a mean AFC of 19.2 and 22.0, respectively.

The mean serum FSH level was 6.7 IU/l (SD 5.5) and ranged from 1.0 to 83.6 IU/l. A total of 19 participants had FSH values over the reference (≥10 IU/l), and two of these had levels >80 IU/l. Mean serum LH was 5.8 IU/l (SD 4.5) and ranged from 0.2 to 38.8 IU/l; mean serum oestradiol was 0.1 nmol/l (SD 0.1) and ranged from 0.02 to 1.1 nmol/l; mean serum SHBG was 55.5 nmol/l (SD 25.0) and ranged from 12.0 to 160.0 nmol/l. The mean serum testosterone level was 1.1 nmol/l (SD 0.4) and ranged from 0.2 to 3.1 nmol/l. The mean free androgen index (FAI) was 0.2 (SD 0.2) and ranged from 0.04 to 2.0. A total of 15 participants had an FAI ≥0.6 (reference value <0.6), indicating biochemical hyperandrogenism. No information was available on clinical hyperandrogenism.

Table 2 presents background characteristics for examined participants (N = 492) and for MoBa daughters who answered the Global Questionnaire (N = 8146) and were not examined. The participants who only completed the questionnaire were born between 1999 and 2005 and were aged 18–24 years. Examined participants’ birth year also ranged from 1999 to 2005, and their age ranged from 18 to 22 years. At the 5% level of statistical significance, there was no difference between the groups in terms of childhood socioeconomic status, parity, height, BMI, or age at menarche. The differences in age, gestational length, birth weight, and current body weight were small, but statistically significant.

**Table 2.** Comparison of background characteristics between MoBa daughters in the current study and MoBa daughters answering the Global Questionnaire.

		Current study		Global Questionnaire		
Clinical characteristics	Measure unit	n	Mean (95% CI)	n	Mean (95% CI)	*P*-value
**Age**	years	492	19.1 (19.0**–**19.2)	8146	19.8 (19.8**–**19.9)	<0.05^1^
**Gestational length**	days	491	279.9 (279.0**–**280.8)	8110	277.2 (276.9**–**277.5)	<0.05^1^
**Birth weight**	gram	492	3597.5 (3549.6**–**3645.4)	8141	3481.7 (3468.3**–**3495.1)	<0.05^1^
**Age at menarche**	years	487	13.0 (12.8**–**13.1)	7712	12.9 (12.9**–**12.9)	0.24^2^
**Height**	cm	485	168.5 (168.0**–**169.0)	8041	168.2 (168.0**–**168.3)	0.26^2^
**Weight**	kg	485	67.7 (66.5**–**68.9)	7802	66.4 (66.1**–**66.7)	0.03^1^
**BMI**	kg/m^2^	485	23.8 (23.4**–**24.2)	7790	23.5 (23.4**–**23.6)	0.08^2^
**Socioeconomic status** ^4^		476		7565		0.27^3^
<High school			10 (2.1%)		211 (2.8%)	
High school			107 (22.5%)		1960 (26.0%)	
Up to 4 years of college			203 (42.6%)		3053 (40.3%)	
>4 years of college			156 (32.8%)		2341 (31.0%)	
**Maternal parity** ^5^		492		8146		0.06^3^
Para 0			195 (39.6%)		3625 (44.5%)	
Para 1			183 (37.2%)		2850 (35.0%)	
Para 2			95 (19.3%)		1268 (15.6%)	
Para ≥3			19 (3.9%)		403 (5.0%)	

1 Welch’s *t*-test.

2 Two-sided *t*-test.

3 Pearsons chi-square test.

4 Information derived from MoBa mothers and fathers answering a questionnaire during pregnancy about highest completed or ongoing education level.

5 Information derived from the Medical Birth registry of Norway: mothers’ parity at the time of recruitment to the MoBa study.

Supplementary Table S1 compares biochemical and clinical variables between participants with and without PCOM. We identified 203 participants with PCOM. Only 50 participants with PCOM were ≥18 and ≥8 years post-menarche (50/471 = 10.6%). We found that age at menarche, haemoglobin A1c, waist-to-hip ratio, and BMI did not differ significantly between the two groups. Menstrual cycle regularity differed significantly along with the biochemical measures of serum AMH, testosterone, FAI, oestradiol, LH, and FSH, while SHBG levels were similar in these two groups.

## Discussion

In this population-based sample of 492 young women aged 18–22 years who had not used hormonal contraceptives for at least two menstrual cycles prior to examination, we found a considerable variation in functional ovarian anatomy. The large IQR (4.7–8.6 cm^3^) of the ovarian volume indicates that even in a similarly aged population-based cohort of young women examined when their ovaries are as large as they will ever be, there is considerable inter-individual variation in ovarian volume. Previous studies with fewer participants in the same age range as in our study indicate that ovarian volume peaks at 7.2–7.7 cm^3^ at around 20 years of age (Kelsey *et al.*, 2013; Gilligan *et al.*, 2019). As in previous studies, we observed that the left ovaries are typically smaller than right ones (Jokubkiene *et al.*, 2012; Korsholm *et al.*, 2017; Abrahami *et al.*, 2020). The side difference is not likely a result of measurement-error (Perven *et al.*, 2014), and two hypotheses to explain this have been put forward. One is embryological, supported by a study of aborted human foetuses that showed larger right ovaries already at early stages of development (Mittwoch and Kirk, 1975). The other is anatomical, based on the difference in venous drainage between the left and the right ovaries, which in males has the well-known clinical corollary of the left testicle as a predilection site for varicocele (Jokubkiene *et al.*, 2012). Regardless of the underlying cause, the functional effects of the side difference is still debated (Abrahami *et al.*, 2020), with some data, primarily from animal studies, suggesting that right-side ovaries ovulate more frequently than those on the left (Ginther, 2020). A larger right ovarian volume could indicate a bigger pool of activated follicles there, and in this study, we also found a significantly higher AFC on the right compared to the left ovaries, as have previous studies (Korsholm *et al.*, 2017). Other studies have found that ovarian volume correlates with AFC and serum AMH, and antral follicles are considered the main source of circulating serum AMH levels (Bentzen *et al.*, 2013; Moolhuijsen and Visser, 2020). This is consistent with our findings, and Fig. 3 shows that AFC and serum AMH levels increase in tandem with the ovarian volume.

Some participants had large ovaries with numerous follicles. According to the Rotterdam consensus, one criterion for diagnosing PCOS in adults is the presence of ≥20 follicles (2–9 mm) in either ovary, or at least one ovary with a volume exceeding 10 cm³, i.e. PCOM. In our study, 127 (127/471 = 27.0%) participants had at least one ovary >10 cm^3^, and 184 (184/470 = 39.1%) participants had ≥20 follicles of 2–10 mm in at least one ovary. A total of 66 (66/470 = 14.0%) had both. The 2023 international guideline on PCOS (Teede *et al.*, 2023) states that ‘adolescents who have features of PCOS, but do not meet diagnostic criteria, an ‘increased risk’ could be considered and reassessment advised at or before full reproductive maturity, 8 years post-menarche’, and further that concerning PCOM ‘the data in young women with a gynaecological age of <8 years (<8 years after menarche) are inadequate’. In the current study, the participants’ mean age at menarche was 13.0 years, hence they are transitioning into the period of reproductive maturity at the time of examination. We found in our study that 43.1% of the study population had PCOM. This underscores the importance of not diagnosing women with PCOS before they have reached reproductive maturity as PCOM can be regarded as a frequent feature in early adulthood.

Other participants had small ovaries, as 127 women had a total ovarian volume of <10 cm^3^, and 25 of these had total ovarian volume of <6 cm^3^. The physiological and clinical implications of small ovaries in early adulthood are under-researched, and ovarian volume is not included in the diagnostic criteria for diminished ovarian reserve by neither the American Society for Reproductive Medicine (Penzias *et al.*, 2020), ESHRE in their BOLOGNA criteria (Ferraretti *et al.*, 2011), nor in the often cited POSEIDON criteria (Alviggi *et al.*, 2016). Ovarian volume was not considered of importance in the diagnostical work-up for primary ovarian insufficiency, a condition that can result from either follicle dysfunction or follicle depletion (Nelson, 2009). Nevertheless, two early studies of infertile patients of various ages indicated low ovarian response to gonadotrophin stimulation in patients with mean ovarian volume <3 cm^3^ (per ovary) (Lass *et al.*, 1997; Sharara and McClamrock, 2001), probably reflecting low AFC in these patients. However, in our data, those with either a left or right ovary 1 SD smaller than the mean had normal serum levels of AMH and total AFC.

There could be various aetiologies behind the observed inter-individual variation in ovarian volume, AFC, and serum levels of AMH. Given that the highest number of oocytes in the ovary occurs during foetal development (Telfer *et al.*, 2023), maternal influences during pregnancy are likely determinants of the embryo’s oocyte pool. However, caution is warranted when discussing oocyte pool, ovarian volume, AFC, and AMH together, as some data have shown a low correlation between serum AMH and density of primordial and primary follicles (von Wolff *et al.*, 2020). The impact of early life factors on AFC and serum levels of AMH later in life is not well understood (Bentzen *et al.*, 2013; Dior *et al.*, 2021), but there seems to be some influence from modifiable risk factors during childhood and adolescence such as neighbourhood poverty or exposure to phthalates on aspects of functional ovarian anatomy later in life (Cinzori *et al.*, 2023; Pan *et al.*, 2024). Ovarian volume correlated with BMI in one study of adolescent girls (Radivojevic *et al.*, 2014). One previous longitudinal study showed that serum AMH in adolescence correlated with serum AMH in puberty and mid-childhood (Hagen *et al.*, 2023), but the putative association between markers of pubertal maturation, such as age at menarche, and later functional ovarian anatomy is not well established (Schuh *et al.*, 2019).

Regardless of aetiology, the physiological and clinical consequences of inter-individual variation in functional ovarian anatomy in early adulthood could be multiple, as ovarian function at this age is assumed to be associated with several health outcomes later in life, such as fertility and perhaps age at menopause. Regarding menopause, our data show that among regularly cycling women in early adulthood, the age where ovarian volume, AFC, and AMH are at peak levels, some women start their reproductive life with smaller ovaries and lower AMH and AFC than others. In this regard, it is worth noting how two longitudinal studies indicated that the rate of decline in AMH differs according to initial high or low serum AMH levels (de Kat *et al.*, 2016; Gohari *et al.*, 2016). On the other hand, recent evidence shows that neither single AMH measurements (Nelson *et al.*, 2023) nor AMH trajectories (de Kat *et al.*, 2019) adds significant precision to models that aim to predict menopause, except in the few cases where primary ovarian insufficiency is diagnosed early. A more complete record of the functional ovarian anatomy as presented in the current study could be informative in this regard. As for fertility, the notion that AMH levels are informative for individual women seeking advice on family planning is not supported by recent high-quality studies, although such use remains widespread (Steiner *et al.*, 2017; Copp *et al.*, 2024). Perhaps reassuringly, both fertility and fecundity are more complex traits than can be measured by a single blood test. Fertility and fecundity involve intricate decisions taken by the individual to maximize reproductive fitness (Sear *et al.*, 2016), and are influenced by reproductive behaviour as well as endocrinological (Jennings and de Lecea, 2020; Mills *et al.*, 2025) and genetic (Venkatesh *et al.*, 2025) underpinnings. The comprehensive data on inter-individual variation in ovarian functional anatomy presented here suggest that the ovary should be considered a modulator in determining these complex reproductive traits, though our current analyses do not allow conclusions as to how.

One strength of this study was that participants were recruited from a population-based sample and were representative of the larger MoBa cohort. Participants’ background characteristics did not differ significantly from non-participating MoBa daughters of similar age. The first generation of MoBa participants are, on average, more highly educated than the general Norwegian population (Nilsen *et al.*, 2009; Vejrup *et al.*, 2022). This could introduce selection bias, as the second generation studied herein may be so too. We assessed the socioeconomic status of participants in the current study and compared it to the broader second-generation MoBa daughter population. The difference in socioeconomic status was not statistically significant. We compared maternal parity at the time of pregnancy and found that it did not differ between the two MoBa populations. Although the participants in our study were representative of the larger MoBa daughter population, our findings may not be generalizable to populations of different ethnicities than Northern Europeans.

No participants were using hormonal contraceptives at the time of examination. This is a notable advantage because previous studies have shown that ovarian reserve markers differ between users and non-users of hormonal contraceptives (Bentzen *et al.*, 2012). Furthermore, selection of participants was not based on the absence or presence of disease, and all examinations were done exclusively in the setting of a scientific study. This is another notable strength, since some previous studies on ovarian functional anatomy included women who were candidates for cryopreservation due to illness (Sermondade *et al.*, 2019; Wakimoto *et al.*, 2020), or who were considerably older or infertile (Marsh *et al.*, 2016; Zhou *et al.*, 2021).

One limitation of this study is the quite low participation rate. With study clinics in only two locations, practical difficulties could arise as many potential participants live far away from these clinics and invitations were sent out to MoBa daughters in all of Norway. The intimate nature of the examinations could have discouraged some invited women from participating, as could the requirement to show up fasting in the morning. Also, the requirement to not use hormonal contraceptives for at least two menstrual cycles, as well as the necessity for the examination to occur on specific cycle days, possibly conflicting with studies or work, could limit participation.

## Conclusion

In this population-based cohort of young women, aged 18–22 years old, who were not using hormonal contraceptives and had their functional ovarian anatomy examined on Days 2–5 during their menstrual cycle, we found a notable inter-individual variation in functional ovarian anatomy. We also found a left-right difference in ovarian volume and AFC, and a moderate positive correlation between ovarian volume, AFC, and serum levels of AMH.

## Supplementary Material

hoaf057_Supplementary_Data

## Data Availability

Restrictions apply to the availability of some, or all data generated or analysed during this study to preserve patient confidentiality or because they were used under license. The corresponding author will, on request, detail the restrictions and any conditions under which access to some data may be provided.
