# Supplementary material for: The functional ovarian anatomy of 492 women aged 18–22 years: a population-based study in Norway
Source: Hum Reprod Open. 2025 Sep 24;2025(4):hoaf057. doi: 10.1093/hropen/hoaf057 (PMC12478698; doi:10.1093/hropen/hoaf057)
Supplement: hoaf057_Supplementary_Data [file hoaf057_supplementary_data.docx]

**Supplementary Table S1. Comparison of participants with (N=203) and without (N=268) polycystic ovarian morphology.**

|  |  | **Participants with PCOM^1^** | | **Participants without PCOM** | |  |
| --- | --- | --- | --- | --- | --- | --- |
| **Clinical characteristics** | **Measure unit** | ***n*** | **Mean (95% CI)** | ***n*** | **Mean (95% CI)** | ***p*-value** |
| **Age at menarche** | years | *202* | 13.0 (12.8–13.2) | *264* | 13.0 (13.0–13.4) | 0.97^2^ |
| **Haemoglobin A1c** | mmol/mol | *176* | 33.8 (33.2–34.4) | *245* | 34.0 (33.3–34.5) | 0.83^3^ |
| **Waist-to-hip ratio^4^** |  | *203* | 0.76 (0.76–0.77) | *268* | 0.76 (0.76–0.77) | 0.97^2^ |
| **Body mass index** | kg/m^2^ | *203* | 22.9 (23.4–24.6) | *268* | 23.8 (23.3–24.3) | 0.61^2^ |
| **Menstrual cycle length**  <24  24–35  >35 | days | *203* | 11 (5.4 %)  142 (70.0%)  50 (24.6%) | *268* | 15 (5.6%)  217 (81.0%)  36 (13.4%) | <0.01^5^ |
|  |  |  |  |  |  |  |
| **Biochemical measures** | **Measure unit** | ***n*** | **Mean (95% CI)** | ***n*** | **Mean (95% CI)** | ***p*-value** |
| **Anti-Müllerian Hormone** | mmol/L | *177* | 37.3 (34.5–40.1) | *234* | 20.1 (18.8–21.5) | <0.01^3^ |
| **Follicle stimulating hormone** | IU/L | *179* | 6.0 (5.8–6.3) | *247* | 6.9 (6.3-7.6) | 0.01^3^ |
| **Luteinizing hormone** | IU/L | *179* | 6.6 (6.0–7.4) | *247* | 5.1 (4.7–5.6) | <0.01^3^ |
| **Oestradiol** | nmol/L | *178* | 0.2 (0.1–0.2) | *247* | 0.1 (0.1–0.1) | <0.01^3^ |
| **Testosterone** | nmol/L | *179* | 1.2 (1.1–1.3) | *247* | 1.0 (1.0–1.0) | <0.01^3^ |
| **Sex hormone binding globulin** | nmol/L | *179* | 55.5 (51.5–59.4) | *247* | 56.0 (53.0–59.1) | 0.82^2^ |
| **Free androgen index^6^** |  | *179* | 0.3 (0.2–0.3) | *247* | 0.2 (0.2–0.2) | <0.01^3^ |
|  |  |  |  |  |  |  |

^1^Polycystic ovarian morphology (PCOM) defined as at least one ovary with >=20 follicles 2-10 mm in size and/or at least one ovary >=10 cm^3^. ^2^Two-sided t-test. ^3^Welch t-test. ^4^Waist-hip ratio: calculated by dividing waist circumference in cm by hip circumference in cm. ^5^Chi square test. ^6^Free androgen index = testosterone/sex-hormone binding globulin
